# A new species of aposematic grasshopper of the genus *Pseudoutanacris* (Acrididae: Gomphocerinae) from the Andean cloud forest of the Ecuadorian Amazon basin

**DOI:** 10.7717/peerj.20376

**Published:** 2026-01-06

**Authors:** Felipe Campos-Yánez, Ana B. García-Ruilova, Diego J. Inclán

**Affiliations:** 1 Instituto Nacional de Biodiversidad, Quito, Pichincha, Ecuador; 2 Facultad de Ciencias Agrícolas, Universidad Central del Ecuador, Quito, Pichincha, Ecuador

**Keywords:** Amblytropidiini, *Pseudoutanacris grilla*, *Megacheilacris graminicola*, Aposematic coloration, Grasshopper

## Abstract

We have identified a new grasshopper species belonging to the genus *Pseudoutanacris* Jago, 1971, in the montane forests of the eastern Andes in Ecuador. This discovery expands the known distribution of the genus, previously limited to a single species in the Bolivian tropics, by over 2,000 kilometers. For the first time, a female of the genus is described, and notes on the ecology and natural history of the species are presented. We also provide the first barcodes of the genus *Pseudoutanacris* Jago, 1971. The males of a newly described species, *Pseudoutanacris grilla* sp. nov. shares a striking coloration pattern with their Bolivian congener,* Pseudoutanacris chromobapta* Jago, 1971, setting them apart from other members of the tribe Amblytropidiini. However, the females maintain a cryptic coloration pattern, similar to that of the tribe members, and display different behavior from the males. During our study, we also observed *Ps. grilla* sp. nov. on the same plant as *Megacheilacris graminicola* (Descamps & Amédégnato, 1971) (Bactrophorinae: Romaleidae), a species with similar chromatic characteristics. This finding also marks the first formal documentation of the new geographical records of *M. graminicola* (Descamps & Amédégnato, 1971) in Ecuador.

## Introduction

Despite being insects of relatively large size, striking colors, interesting shapes, and economic importance in agriculture, the Orthoptera group has been relatively understudied in the neotropics, and Ecuador is no exception. With a total of approximately 30,000 species known worldwide, of which 40% correspond to short-horned diurnal grasshoppers (Caelifera), the country’s diversity is only about 2% of the total (Cigliano et al., 2025). In well-known groups such as vertebrates, plants, butterflies, or certain families of beetles, the percentage of diversity reaches values greater than 5% (Campos, 2020). This disparity highlights the limited research efforts directed towards orthopterans, particularly in a diverse country like Ecuador, where vast areas such as the Páramos and montane forests of the Andes remain largely unexplored.

The Amblytropidiini tribe, mainly distributed in the Neotropical region, is one of the 19 recognized tribes of the Gomphocerinae subfamily (Acrididae) found almost worldwide, except in Oceania and certain circumpolar areas. Amblytropidiini is formed by nine genera and about 39 species (Cigliano et al., 2025). In Ecuador, the presence of the tribe is limited to just two species: *Fenestra platyceps* (Hebard, 1924) and *Peruvia nigromarginata* (Scudder, 1875; Buzzetti & Carotti, 2008; Cigliano et al., 2025). This low diversity value likely indicates undersampling, suggesting that the country’s biodiversity of this group may increase in the future.

The genus *Pseudoutanacris* Jago, 1971 is currently composed of a single described species found in the tropical zone southeast of Bolivia, in the Department of Santa Cruz de la Sierra. Other photographic records extend its distribution to the southeast of Peru, in the Department of Madre de Dios (Bay, 2015). At first glance, *Pseudoutanacris* differs from all species of the tribe Amblytropidiini and even from the subfamily Gomphocerinae by its coloration, which is composed of bright tones and striking alternating colors, unlike the brown, cream, and orange tones that dominate in other species of the group (Jago, 1971). The type species of the genus, *Pseudoutanacris chromobapta* (Jago, 1971), is known only from males, and its name reflects the colorful nature of *Utanacris pulchra* Miller, 1934, a member of the subfamily Catantopinae from the Malay Peninsula.

In this publication, we aim to share the discovery of a new species of acridid grasshopper, preliminarily identified by Gray Catanzaro as a member of the tribe Amblytropidiini through the digital platform iNaturalist. Simultaneously, we acknowledge the importance and utility of this tool, which continuously contributes to the advancement of global biodiversity knowledge.

This study marks the beginning of a series on the acridoid grasshoppers of Ecuador. The National Institute of Biodiversity of Ecuador has been investigating these grasshoppers for the past years, building on the research conducted by a French mission from the Museum of Natural History of Paris in the late 20th century.

## Materials & Methods

All the specimens cited in this work are deposited in the scientific collection of invertebrates at the National Biodiversity Institute (INABIO) in Ecuador, representing the MECN (Ecuadorian Museum of Natural Sciences) collections. Labels of type material are quoted separately, line breaks are indicated by a backslash (/), and additional information is given between brackets ([ ]).

The specimens studied were collected under research permits MAAE-DBI-CM-2022-0228 and MAATE-DBI-CM-2023-0309, issued by the Ministry of Environment of Ecuador.

Specimens of the new species were compared with published descriptions of *Ps. chromobapta* and with photographs of the type specimen (MNHN-EO-CAELIF1175) available in the Orthoptera Species File (Cigliano et al., 2025). Measurements of the study material (holotype and paratypes) were taken with a digital caliper (accuracy ± 0.1 mm). Total body length refers to the insect’s body length from the head to the tip of the abdomen. Head width was measured between the widest points of the head, the eyes, in the case of males; in females, it was measured from the posterior edge of the head. Head length was measured from the tip of the fastigium to the posterior edge of the occiput along the midline carina. Pronotum length was measured from its anterior to posterior edge along the midline. Tegmina length was measured from their base (insertion into the thorax) to the apex. Hind femur length was measured from the trochanter to the apex of the femur (knee). The measurement of the antenna includes the entire antennal organ.

The male genitalia were prepared following the procedure proposed by Hubbell (1932). The process begins by softening the entire animal in hot water for a short period (30 s) and focusing on the tip of the abdomen for a slightly longer time. Using a stereo microscope and scalpel, an incision was made on the left side between the distal tergites and sternites. The membrane connecting the ventral surfaces of the paraprocts to the sclerotized plate at the cephalic end was then cut. The caudal end of the genital mass was carefully slid outward to expose the penis. The pallium is separated from the subgenital plate, and the entire genital apparatus is removed. A treatment with 10% KOH in a heated water for 2 min is performed to study the genitals, and was then stabilized with 1% acetic acid and washed in distilled water. If necessary, soft tissues are cleaned, and the structures are preserved in 70% alcohol. A Zeiss Stemi 2000-C microscope and a Canon G10 camera were used for the genital study. Photographs of live animals were captured using a Nikon D3300 camera with a Nikkor 105 mm macro lens and a Sony Alpha7 camera with a Sigma 105 mm macro lens.

To ensure whether the male and female specimens belonged to the same species and to validate its taxonomic position, we amplified the classical animal DNA barcode, a fragment of the mitochondrial cytochrome oxidase I (COI) gene from three specimens of the new species (two males MECN-FC1987, MECN-FC1992, and one female MECN-FC1988) and one more of *Pe. nigromarginata.* DNA extraction was performed from the hind leg muscle of the specimens, in collaboration with the Canadian Centre for DNA Barcoding (CCDB), and using the C_LepFolF and C_LepFolR primers. Comprehensive technical details of the extraction, amplification, PCR, and sequencing protocols can be found in Wilson (2012). Also, we used about 30 sequences available in GenBank and BOLD System from eight species of the tribe Ablitropidiini, with 400 to 659 bp approximately from the COI gene. The sequences correspond to different countries since there are no public sequences in Ecuador or neighboring countries. We provide for the first time barcodes of the genus *Pseudoutanacris*.

To delimit the species, the Barcode Gap Analysis and Distance Summary were generated in BOLD Systems based on Kimura’s two-parameter distances and default settings. They were visualized and edited using MEGA XI software. In addition, we used online ABGD analysis as another distance-based method (Puillandre et al., 2012), using the K2P model and, since the program is optimized for the COI gene, the default values of Pmin = 0.001 and Pmax = 0.10, steps = 20, and Nb bins = 20 were used. We also used a value of relative gap width (X) of 0.75 to increase the sensitivity of the analysis (Puillandre et al., 2012). For this analysis, we used the K2P model because it is used in other barcoding studies (*e.g.*, Hendrich et al., 2015; Hawlitschek et al., 2016). Sequences are available in GenBank under accession numbers PV173915, PV173916, PV173917, PV173917, PV173918, and in the BOLD Systems database (http://www.boldsystems.org/) under the public dataset DS-GRILLA: “A new Ecuadorian species of the genus Pseudoutanacris and other public barcodes of the tribe Ablitropidiini” (DOI: http://dx.doi.org/10.5883/DS-GRILLA).

The electronic version of this article in Portable Document Format (PDF) will represent a published work according to the International Commission on Zoological Nomenclature (ICZN), and hence the new names contained in the electronic version are effectively published under that Code from the electronic edition alone. This published work and the nomenclatural acts it contains have been registered in ZooBank, the online registration system for the ICZN. The ZooBank LSIDs (Life Science Identifiers) can be resolved and the associated information viewed through any standard web browser by appending the LSID to the prefix http://zoobank.org/. The LSID for this publication is: (urn:lsid:zoobank.org:pub:0237C921-6F8E-44E6-A97B-27FB32C37E28). The online version of this work is archived and available from the following digital repositories: PeerJ, PubMed Central SCIE, and CLOCKSS.

## Results

**Table:** 

**Taxonomy**
**Family Acrididae MacLeay, 1821**
**Subfamily Gomphocerinae Fieber, 1853**
**Tribe Amblytropidiini Brunner von Wattenwyl, 1893**
**Genus *Pseudoutanacris*Jago, 1971**

**Table:** 

**Figure 1:**
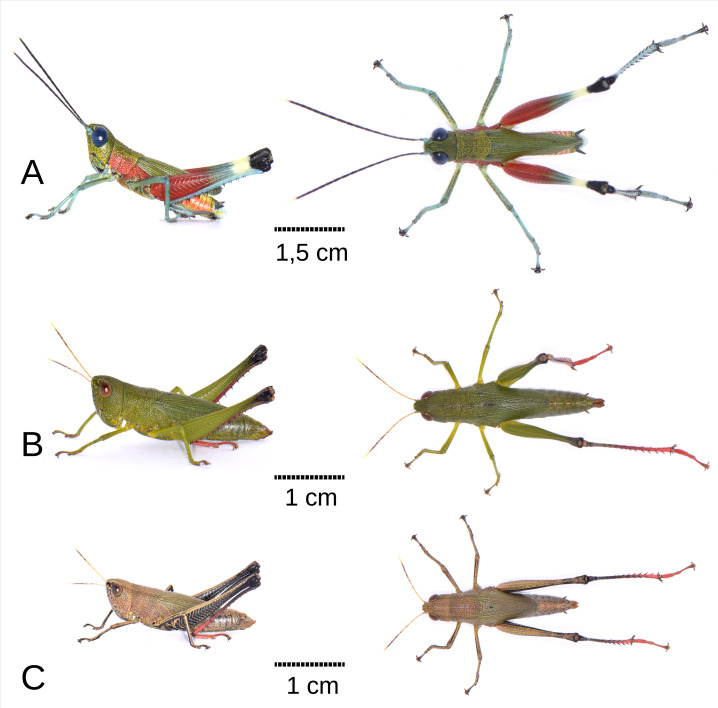
Habitus of *Pseudoutanacris grilla* sp. nov. (lateral and dorsal views). (A) Male Holotype MECN-FC-2309. (B) Female Allotype MECN-FC-2310 (green chromotype). (C) Female Paratype MECN-FC-2311 (brown chromotype). Photographs by F. Campos.

**Figure 2:**
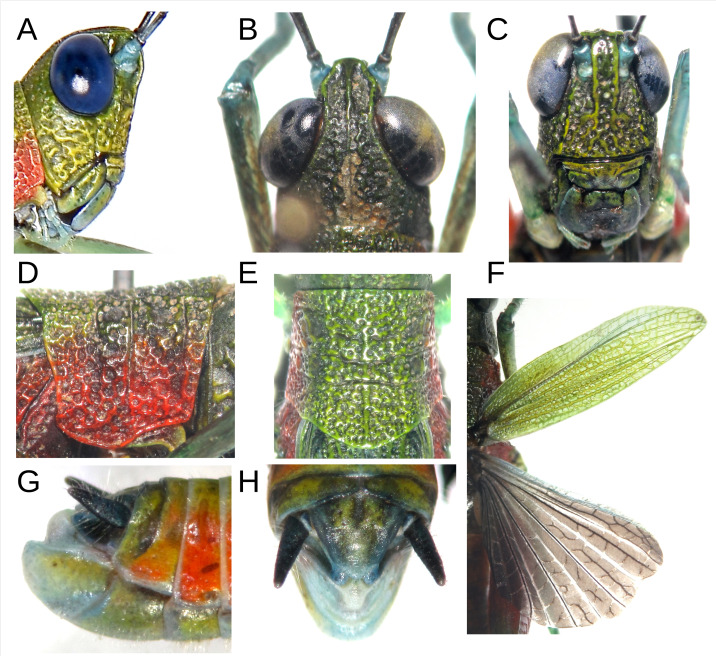
Anatomical characteristics of male holotype MECN-FC-2309 and Paratype MECN-FC-2308. (A) Head, lateral view. (B) Same, dorsal view. (C) Same, frontal view. (D) Pronotum, lateral view. (E) same, dorsal view. (F) Anterior and posterior wings, dorsal view of MECN-FC-2308. (G) Terminalia, lateral view. (H) Same, superior view. Photographs by F. Campos.

**Figure 3:**
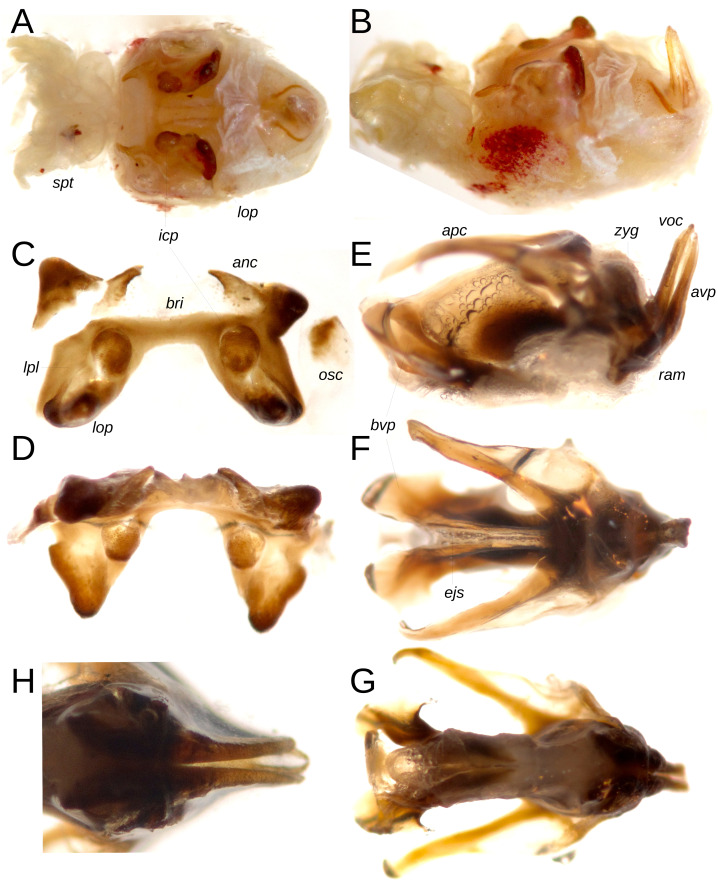
Internal genitalia of male Paratype MECN-FC-1692. (A) Axial view of the genital mass with its soft structures. (B) Same, lateral view. (C) Epiphallus, axial view. (D) Same, anterior view. (E) Phallic complex, lateral view. (F) Same, dorsal view. (G) Same, ventral view. (H) Ramus and apical valve of penis, infero-posterior view. Key to terms: bridge (bri), ancora (anc), oval sclerite (osc), lophus (lop), lateral plate (lpl), internal conical protuberance (icp), apodeme of cingulum (apc), zygoma (zyg), valve of cingulum (voc), apical valve of penis (avp), basal valve of penis (bvp), spermatic tubes (spt), ejaculatory sac (ejs), ramus (ram). Photographs by F. Campos.

**Figure 4:**
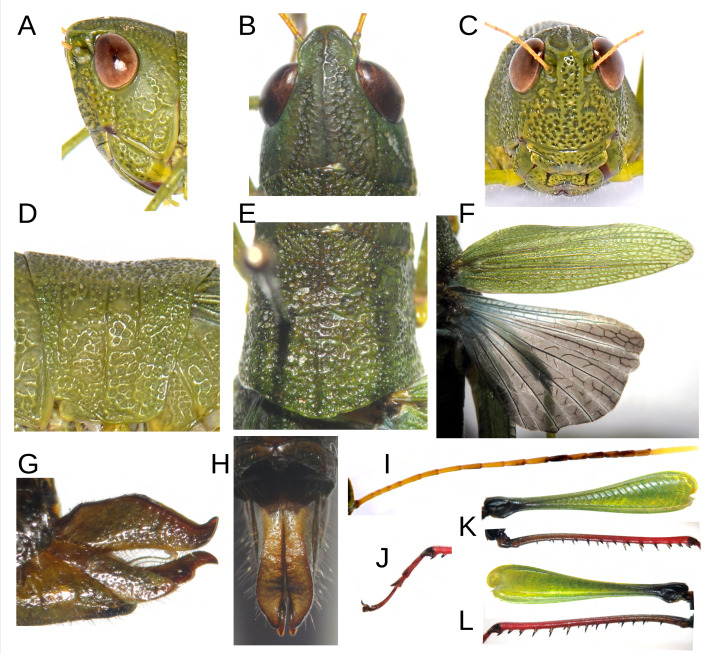
Anatomical characteristics of female Allotype MECN-FC-2311. (A) Head, lateral view. (B) Same, dorsal view. (C) Same, frontal view. (D) Pronotum, lateral view. (E) Same, dorsal view. (F) Tegmina and hind wing. (G) External genitalia, lateral view. (H) Same, dorsal view. (I) Antenna. (J) Tarsus of posterior leg. (K–L) External and internal side of the hind leg. Photographs by F. Campos.

**Figure 5:**
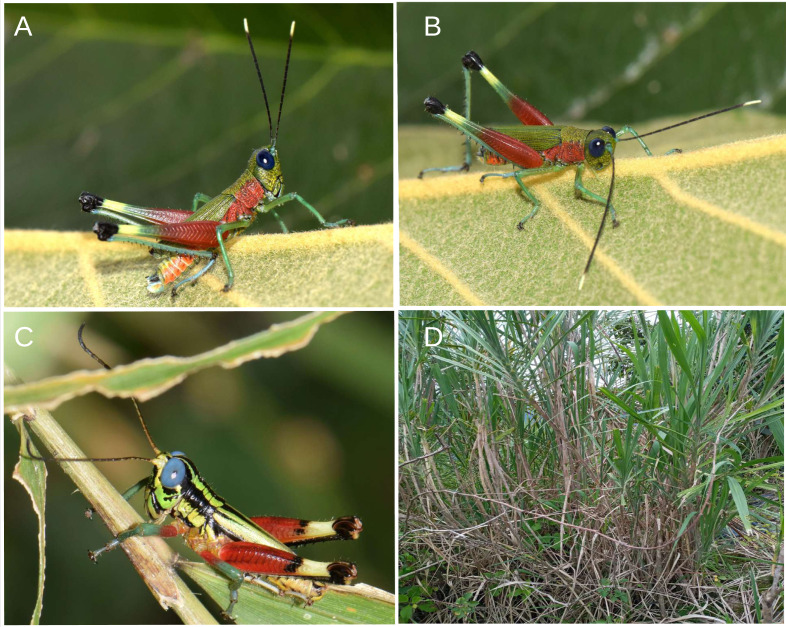
Related species of acridoidea in their natural habitat. (A–B) *Pseudoutanacris grilla* sp. nov. (male). (C) *Megacheilacris graminicola* (Descamps & Amédégnato, 1971). (D) Natural habitat of *P. grilla* sp. nov. Photographs by F. Campos.

***Pseudoutanacris grilla* **sp. nov.****
(Figs. 1–5)
https://zoobank.org/NomenclaturalActs/1ee26cd7-7b6f-4894-8971-374cf07f9214

Type material

Holotype: “♂ Ecuador. Morona Santiago,/El Tigrillo, road Macas-/Guamote 1,820 m./−2,217458, −78,224425/13-may-2025 F. Campos & J. Granizo”; “[Depository:] MECN-FC-2309”; “[red label] HOLOTYPE/*Pseudoutanacris grilla/* Campos, F.”.

Allotype: “♀ Ecuador. Morona Santiago,/El Tigrillo, road Macas-/Guamote 1,820 m./−2,217458, −78,224425 / 13-may-2025 F. Campos & J. Granizo”; “[Depository:] MECN-FC-2310”; “[red label] ALLOTYPE/*Pseudoutanacris grilla/* Campos, F.”.

Paratypes: 5♂, 1♀: Ecuador. Morona Santiago,/El Tigrillo, road Macas-/Guamote 1,820 m./−2,217458, −78,224425/25-ago-2023 F. Campos”; “[Depository:] MECN-FC-1691-4, MECN-FC-1687-8”; “[yellow label] PARATYPE/*Pseudoutanacris grilla/* Campos, F.”; “DNA voucher specimen/CCDB Lab code/Process ID/ORTEC164-24, ORTEC160-24, ORTEC168-24”. 2♂, 1♀, 1♀ (nymph): Same data as Allotype; “[Depository:] MECN-FC-2307-8, MECN-FC-2311-2”; “[yellow label] PARATYPE/*Pseudoutanacris grilla/* Campos, F”.

Etymology

The word “grilla” is derived from the Spanish word “grillo,” which refers to the female of an orthoptera species known as “grillo” (genus *Gryllus*) and taxonomically belongs to the infraorder Gryllidea. In Ecuador, most orthoptera are commonly referred to as “grillos” (crickets). The term is also used in Ecuadorianism to describe a person who seeks attention and exhibits behavior similar to that of the species. This name will be treated as an arbitrary combination of letters.

Description

Male. Small to medium-sized insect with quite rough tegument.

Coloration: The insect exhibits a predominantly green and red color pattern (Fig. 1A). The head, pronotum disc, and wings are olive green, while the face also shows yellowish tones (Figs. 1A; 2A–2C). Red coloration is visible on the proximal half of the posterior femurs, as well as on the sides of the thorax and pronotum (Figs. 1A; 2D–2F). The eyes appear blue in life (Figs. 1A, 2A). The antennae are black with a cream-colored apex and light blue scape and pedicel (Figs. 1A; 2A–2C). The tibiae and tarsi of all legs are a faint turquoise, with the femurs of the front and middle legs displaying a jade green hue with brown flecks. The distal half of the posterior femurs has two faint green and cream bands near the black knee (Fig. 1A). The abdomen is orange on the sides and yellowish dorsally (Figs. 1A; 2G). Ventrally, the entire body is light olive green with scattered yellowish tones. The cerci are black, and the posterior tip of the abdomen features light blue, white, and yellow tones (Figs. 2G–2H). The hindwings have a smoky coloration with the front edge light blue (Fig. 2F).

Head: Slightly wider than long with prominent, almost oval eyes. The frontal costa is 1.5 times the width of the antennal scape and extends from the tip of the fastigium to below the median ocellus, for a distance smaller than the width of the antennal scape, and then disappears. Along its length, it is marked by small but deep subcircular points, arranged into two parallel rows somewhat disordered (Fig. 2C). The fastigium of the vertex is truncated in front, with strong lateral and middle carinae, the latter extending moderately along the entire occiput (Fig. 2B). The antennae size ranges from 1.9 to 2.2 times the combined size of the head and pronotum (*n* = 8), with 22 segments slightly narrower at their proximal end.

Thorax: The pronotum is rough on the dorsal and lateral surfaces, with three moderately marked sulci. The middle carina is evident throughout its entire length, while the lateral carinae are almost absent, except at the posterior end of the metazone. The posterior edge of the pronotum is angular and rounded, with a posterior projection of 25 degrees (Fig. 2D). The lateral lobes have a straight anterior edge, a slightly obtuse lower anterior angle, and a sinuous lower edge that is concave in the prozone and convex in the metazone. The posteroinferior angle is barely obtuse and appears subcircular. The posterior edge is barely sinuous, concave below, and convex at the upper end (Fig. 2D). The tegmina have very marked venation and are abbreviated with an extension that varies between the 8th tergite and the tip of the supra-anal plate. The hindwings are approximately 20% shorter than the tegmina and exhibit an intermediate, incomplete venation with a sinuous appearance (Fig. 2F).

Legs: Long, with middle and forelegs almost as long as the hind femur extension, and the hind femur is $ \frac{1}{4} $ longer than the tip of the abdomen. The hind leg has 10 internal and external tibial spines, with the inner ones approximately twice the size of the outer ones.

Abdomen: The abdomen has robust conical cerci that project directly backward. The triangular epiproct has a slightly rounded posterior angle. In top dorsal view, the subgenital plate is subtriangular with a rounded tip at the posterior end; in lateral view, the apical edge lies in a plane almost parallel to the body axis, showing the pallium projecting upwards (Figs. 2G–2H).

Phallic complex: The genital mass exhibits a subtriangular morphology when viewed dorsally (Figs. 3A, 3B), with the epiphallus and aedeagal valves clearly visible in dorsal and lateral perspectives. The cingulum presents elongated, V-shaped apodemes in axial view; laterally, the terminal portion of the cingulum valves and the apical valve of the penis are thin and markedly extended, oriented nearly perpendicular to the ectophallus (Figs. 3E, 3H). The anterior region of the apical valve of the penis is broad, curved, and heavily sclerotized (Fig. 3E), while the basal segment of the penis valve displays lateral spiniform expansions in dorsal and ventral views (Figs. 3F, 3G). The ramus is short, and the zygoma is well developed (Fig. 3E). The epiphallus exhibits a dorsal ‘H’-shaped configuration, with the ancora spiniform, whose tips are directed medially; the bridge is narrow, short, and straight, connecting the two lateral plates of the epiphallus, which possess strongly invaginated external margins toward the center. Internal conical protuberances are of medium size relative to the lobes, which are notably sclerotized and dorsally expanded (Figs. 3B, 3D).

Female.

The female exhibits a broader overall morphology, particularly in the cephalic and thoracic regions, resulting in a fusiform shape (Figs. 1B–1C; 4B). The size ratio between females and males is approximately 174% (*n* = 3 + 8), with a range fluctuating between 149% and 242% relative to all adult male and female specimens of the type series (Table 1). *In vivo*, coloration displays variability, with at least two distinguishable chromatic morphs: a green form (allotype, Fig. 1B), and a brown form (paratype, Fig. 1C). Constant chromatic features between these two forms include the antennae, not only in terms of coloration (yellow, gray, and mustard) but also in the pattern of spots displayed on the different antennal segments (Fig. 4I); notably, red pigmentation is present on the tarsi and predominantly on the distal sections of the tibiae of the posterior legs (Figs. 1B–1C; 4J–4L). Additionally, in both chromotypes, the knee of the hind legs are black (Figs. 1B, 1C; 4K–4L), and a diffuse dark dorsolateral band is observed primarily on the dorsal portion of the pronotal lobes (Figs. 1B–1C); the wings are membranous and smoky in appearance, with the anterior margin and insertion zone exhibiting a ligth blue coloration (Fig. 4F); and the eyes are brown (Figs. 4A–4C). The green morphotype displays darker shades on the tegmina and yellowish tones along the lateral margins of the body, especially at the level of the coxae of all three pairs of legs and the region corresponding to the mouthparts (Fig. 1B). Conversely, the brown morphotype is characterized by the internal and external surfaces of the posterior femora bearing a black coloration adorned with brown chevrons; the remainder of the body, excluding the tibiae and tarsi of the posterior legs, presents light brown to greenish-brown hues on the tegmina (Fig. 1C).

Anatomically, the female shares most morphological features with the male, with the exception of a sligtht lateral carina extending along the entire length of the pronotum (Fig. 4D). The width of the head in the posterior region is significantly greater in females; this, combined with less voluminous eyes, results in a subequal axial perspective, while in males, the eyes appear much more protruding than the posterior area of the head (Figs. 1B, 4B). The length of the antennae is similar between males (range = 12.6–14.2) and females (range = 10.8–13.7), despite the significant size difference between the sexes (Figs. 1A–1C; Table 1).

### Comparative diagnosis

The Ecuadorian species *Ps. grilla* **sp. nov***.* can be is distinguished from the Bolivian species *P. chromobapta* (the only known species of the genus until now) primarily by its coloration. *Ps. grilla* **sp. nov**. has a green head and red basal half of the posterior femurs and lateral area of the thorax (Figs. 5A–5B), while *Ps. chromobapta* has a blue head, a red band on the second basal quarter of the posterior femurs, yellowish-green sides of the thorax, and a black dorsal-lateral band that extends from the anterior edge of the pronotum to the tip of the tegmina (Fig. 5B).

Anatomically, *Ps. grilla* **sp. nov.** has a rougher integument compared to *Ps. chromobapta*, with deeper punctuations on the thorax and head. The lateral carinae on the pronotum are barely visible in *Ps. grilla* **sp. nov.**, unlike the Bolivian species where they are absent. Additionally, the posterior edge of the pronotum has a slightly more angular shape in the Ecuadorian species. The frontal costa in *Ps. chromobapta* is sulcate, whereas, in *Ps. grilla* **sp. nov.,** it appears punctuated by two parallel lines of consecutive dots. The antennae in males of *Ps. grilla* **sp. nov.** are slightly shorter than the total body length (x = 87%; r = 78–94%; *n* = 8), while in *Ps. chromobapta*, it is slightly longer (105%; *n* = 1). The male terminalia shape, when viewed from the dorsum, is rounded in the Ecuadorian species and more angular in the Bolivian species.

In life, males of the two species of *Pseudoutanacris* typically stand upright with their front legs extended, hind legs poised to jump, and heads raised, displaying their antennae (Figs. 5A–5B). In contrast, females of the newly described species, with have more camouflaged colors, tend to adopt a flattened posture with their legs bent.

**Table 1:** Measurements of *Pseudoutanacris grilla* sp. nov.

	1♀ (nympha) mm	5♂ mm
Head width	5,1	(3,4 –3,5)
Head length	4,9	(2,9 –3,6)
Antenna length	13,7	(13,2 –14,2)
Pronotum length	4,1	(3,0 –3,4)
Tegmina length	12,4	(8,4 –9,8)
Posterior femur length	19,2	(12,7 –13,0)
Total length	33,6	(14,5 –17,2)

### Molecular delimitation

A total of four mitochondrial COI barcode sequences were obtained. The data herein represent the first published DNA barcodes for the genus *Pseudoutanacris* and the start of the reference library of orthoptera barcodes from Ecuador. The result of barcode analysis for specimens MECN-FC1987 (♂), MECN-FC1988 (♀), and MECN-FC1992 (♂) supports that the genetic distance between males and females is 0%. The distribution of intraspecific and interspecific distances shows the relationship of sequenced specimens in the tribe Amblytropidiini (Table 2).

**Table 2:** Summary of the results of the genetic distance between species of the tribe Amblytropidiini.

		1	2	3	4	5	6	7	8
1	*Pseudoutanacris grilla* ** sp. nov**	0							
2	*Amblytropidia mysteca*	13.94	0.5						
3	*Boopedon gracile*	17.68	12.22	1.54					
4	*Peruvia nigromarginata*	15.52	15.36	14.42	0				
5	*Sinipta dalmani*	19.14	13.31	16.11	17.58	0.92			
6	*Sinipta hectorisperonii*	19.98	14.18	16.49	18.75	1.92	0.48		
7	*Syrbula admirabilis*	22.65	15.01	18.97	16.58	18.89	18.75	0.34	
8	*Syrbula montezuma*	18.45	14.45	14.06	16.01	16.52	17.11	10.67	0

### Distribution and habitat

This species is only known from the type locality in the montane forest ecosystem of the Amazonian Andean foothills of the Province of Morona Santiago in the Ecuadorian Amazon. The collection site is a disturbed area dominated by grass, bushes, and remaining patches of forest, which are part of the buffer zone of the Sangay National Park. The exact collection point corresponds to an area with a steep slope and vegetation dominated by tall herbaceous plants, surrounded by shrubs (Fig. 5D).

### Behavior

During field work in 2023, in a small patch of tall grass measuring three to four square meters, we observed around a dozen red grasshoppers perched on the upper leaves of the Kikuyu grass *Cenchrus clandestinus* (Hochst. ex Chiov.), an invasive species from Africa. Upon closer inspection for photography and collection, we identified two different species, *P. grilla* **sp. nov.** and *Megacheilacris graminicola* (Descamps & Amédégnato, 1971) (Romaleidae) (Fig. 5C), both coexisting in the same habitat. The individuals of *Ps. grilla* **sp. nov.** were more spread out compared to those of *Megacheilacris* (Descamps, 1978). When the specimens were collected, found only male individuals of *Pseudoutanacris,* whereas males, females, and juveniles of Megacheilacris were present. Locating *Pseudoutanacris* females was challenging, as they were well camouflaged in the lower part of the vegetation, blending in seamlessly with the dense grass. We were only able to collect one large, freshly molting female, which raised initial doubts about whether it belonged to the same species.

Almost two years later, we returned to the same site for two days and again found only males during the day; however, at night, we were able to collect three females at the highest tips of the grasses. Additionally, we found in another plant, *Gynerium sagitatum* (Aubl.) P. Beauv., 1812 (Poaceae) (Fig. 5D), located less than 10 m away from the first place, a group composed of several males and two females, which displayed an evidently different coloration pattern: a brown chromotype (Fig. 1C) at the lower part of the plants, where dry leaves accumulate, and a green chromotype (Fig. 1B) at the top of the plant, the latter close to several males of the species. In both cases, the female individuals were in a curled and mimetic position with the environment.

These episodes highlight interesting aspects of *P. grilla* **sp. nov.** Firstly, there is the marked sexual dimorphism, which is not only related to size, shape, and color but also to the differentiated behavior between both sexes. While the males are exposed in a characteristic raised position in the upper zone of the vegetation, the brown females hide among the dry and low stems in a flattened position, and green females seem to occur on the green stems of the taller grasses. We believe that the presence of chromotypes adapted to different ecological conditions could express a great evolutionary plasticity of the species.

A second aspect relates to communication, specifically in terms of aposematism, which is present in both sexes in their own way through warning colors. In females, these signals remain hidden until the moment of maximum danger, when they extend their legs and expose the red tibiae that contrast with the hind femurs to deter predators in the foliage. In the case of males, their colors are always evident to aerial predators.

Also related to communication is the presence of antennae of considerable length, adorned with white/yellow at the tip, like a flag, in both sexes. While the general color of the antennae differs between the sexes, their size is almost identical, despite the significant variation in body size between the sexes. This situation suggests that, beyond the sounds produced, the species possesses a complex communication system that integrates acoustic signals with visual and/or tactile signals.

Finally, a very interesting element is the phenomenon of convergent evolution, expressed in a nearly identical aposematic coloration pattern between males of two different species and two distinct evolutionary lineages, which not only share habitat but also food resources. There are undoubtedly several outstanding questions that we would like to clarify in future behavioral studies; however, these are aspects that position this genus and this species as interesting subjects for study in the fields of sexual evolution, adaptability, and interspecific and intraspecific communication.

### New records

**Table:** 

*Megacheilacris graminicola* **(Descamps & Amédégnato, 1971)**
(Fig. 5D)

**Type locality:** Colombia, Departamento de Putumayo, entre El Mirador y pepino, altitud 1.500 m, 1♂ Holotype, 1♀ Allotype, 22♂ and 18♀ paratypes, eight larves, 8-XI-1968. Lg. M. Descaps, E. Lagos, R.Restrepo y H. Salazar. Depository: Museum of Paris.

**New records:** “[♂] Ecuador. Morona Santiago,/M. El Tigrillo, road Macas-/Guamote 1,820 m./−2,217458, −78,224425/25-ago-2023 F. Campos”; “[Depository:] MECN-FC-1700”

“[1♂, 1♀] Ecuador. Napo/Baeza, junto Río Quijos/−0.457873, −77.89381/17-11-2021 1,800 m/Manual F. Campos”; “[Depository:] MECN-FC-0094; MECN-FC-0100”

**Distribution:** Based on collection records and confirmed observations from the iNaturalist (https://www.inaturalist.org/observations?place_id=97389&taxon_id=760233) platform, the distribution of the species corresponds with Piemontane and Lower Montane Forest (457 to 2,000 m) between the Department of Putumayo, Colombia, and the Province of Zamora Chinchipe, in southern Ecuador.

## Discussion

Our study has identified a new grasshopper species, *P. grilla* **sp. nov.**, in the montane forests of the eastern Andes of Ecuador. This discovery expands the known distribution of the genus *Pseudoutanacris*, previously limited to Bolivia, by more than 2,000 kilometers. The finding also suggests that these same species or other as yet undiscovered species could be found in the montane forests of the Peruvian Amazon. The distinctive color pattern of *P. grilla* **sp. nov.**, shared with *Ps. chromobapta,* underscores the uniqueness of this genus within the tribe Amblytropidiini.

*Pseudoutanacris* and *Amblytropidia* Stål, 1873 are closely related, according to Jago (1971) and our genetic analysis (see Table 2). However, they differ not only in their coloration pattern, but also in the marked sexual dimorphism of the species, where, in addition to their shape, size, and color, it is also expressed in a differentiated sexual behavior, in which visual and acoustic communication seem to play an important role. While *Amblytropidia* generally uses low-sized herbaceous habitats, *Ps. grilla* **sp. nov.** takes advantage of a particular habitat consisting of a mosaic of large herbaceous plants and shrubs located on steep slopes. Males of *Ps. grilla* **sp. nov.** use the upper parts of the plants and during their escape jump along the slope and glide a considerable distance, while females apparently remain hidden inside the foliage.

The co-occurrence of *Ps. grilla* **sp. nov.** and *M. graminicola* in the same habitat suggests potential ecological interactions or convergent evolutionary traits, particularly in coloration and behavior. This raises intriguing questions about the adaptive strategies and communication mechanisms of these species, warranting further investigation. Our findings also establish new geographical records for *M. graminicola* in Ecuador, contributing to a better understanding of Orthoptera diversity in the region. Differences in sexual dimorphism and behavior between male and female *Ps. grilla* **sp. nov.** offer insights into their ecological roles and reproductive strategies.

## Conclusions

In conclusion, our research has identified a new species, *Ps. grilla* **sp. nov.,** which significantly expands the known distribution of the genus *Pseudoutanacris*. This discovery highlights the rich biodiversity of the Ecuadorian Andes and emphasizes the importance of ongoing exploration and documentation of Orthoptera in the region.

Our findings shed light on the unique coloration and behavior of *Ps. grilla* **sp. nov*.,*** suggesting potential ecological interactions and convergent evolution with other grasshopper species. The new geographical records for *M. graminicola* further contribute to our understanding of Orthoptera diversity in Ecuador.

Future studies should focus on addressing the limitations of our research by increasing sample sizes, exploring additional habitats, and conducting comprehensive behavioral analyses. These efforts will deepen our knowledge of the ecological roles, adaptive strategies, and evolutionary relationships of these intriguing insects.

## Additional Information and Declarations

### Field Study Permissions

The following information was supplied relating to field study approvals (*i.e.*, approving body and any reference numbers): Field experiments were approved by the Ministerio del Ambiente, Agua y Transición Ecológica del Ecuador (permissions number No MAAE-DBI-CM-2022-0228 and MAATE-DBI-CM-2023-0309).

### Data Availability

The following information was supplied regarding data availability: The sequences are available at GenBank: PV173915, PV173916, PV173917, PV173917, PV173918; and in BOLD Systems: DOI: http://dx.doi.org/10.5883/DS-GRILLA.

### New Species Registration

The following information was supplied regarding the registration of a newly described species: *Pseudoutanacris grilla* species LSID: urn:lsid:zoobank.org:act:1EE26CD7-7B6F-4894-8971-374CF07F9214 Publication LSID: urn:lsid:zoobank.org:pub:0237C921-6F8E-44E6-A97B-27FB32C37E28.
